# Your Lifestyle As Medicine: the impact of a citizen initiative for people with type 2 diabetes using peer coaching and self-management

**DOI:** 10.1136/bmjnph-2025-001362

**Published:** 2025-11-05

**Authors:** Daphne Charlotte Josephine Raad, Anne Marit Koome, Raymond Noordam, Hanno Pijl, David Van Bodegom

**Affiliations:** 1 Department of Public Health and Primary Care, Leiden University Medical Center, Leiden, Netherlands; 2 Leyden Academy on Vitality and Ageing, Leiden, Netherlands; 3 Department of Clinical Epidemiology, Leiden University Medical Center, Leiden, Netherlands; 4 Department of Internal Medicine, Division of Endocrinology, Leids Universitair Medisch Centrum, Leiden, Netherlands

**Keywords:** Diabetes mellitus, Dietary patterns, Metabolic syndrome, Weight management

## Abstract

**Background and aim:**

Sustained lifestyle changes are crucial for the remission of type 2 diabetes (T2D) but remain challenging. Citizen initiatives using peer coaching and self-management may offer a promising alternative to professional medical care. This study evaluated *Your Lifestyle As Medicine* (YLAM), a Dutch citizen initiative for people with T2D. We aimed to assess its impact on metabolic parameters and to examine participants’ engagement.

**Methods:**

This observational study analysed self-reported data on weight, waist circumference, fasting glucose and glycated haemoglobin (HbA_1_c) from participants in YLAM’s online community. Participants could report their self-measured data on a weekly basis. Linear mixed-model analyses, stratified by sex, were used to assess changes in metabolic parameters over time. Additionally, we evaluated participants’ engagement through reporting duration and weekly reporting rates.

**Results:**

We assessed all 232 people with T2D who reported multiple measurements for at least 3 months. The median reporting duration was 11.2 months (IQR 4.6–26.5). Weekly reporting rates were 59% for weight, 55% for waist circumference and 52% for fasting glucose, and 12-weekly reporting rates were 49% for HbA_1_c. Overall, mean weight, waist circumference, fasting glucose and HbA_1_c improved in the first year in both women and men. More specifically, weight decreased by 7.2 kg in women (95% CI –7.6 to –6.8) and by 7.4 kg in men (95% CI –8.0 to –6.8). This represented a mean relative weight loss of 9.0% (SD 7.7) and 8.6% (SD 6.5), respectively. Waist circumference decreased by 8.9 cm in women (95% CI –9.4 to –8.5) and by 8.5 cm in men (95% CI –9.1 to –7.8). Fasting glucose decreased by 1.15 mmol/L in women (95% CI –1.32 to –0.98) and by 0.49 mmol/L in men (95% CI –0.75 to –0.23). HbA_1_c decreased by 14.5 mmol/mol in women (95% CI –17.4 to –11.6) and by 9.1 mmol/mol in men (95% CI –13.2 to –5.0). Of all participants, 44% reported data for longer than a year and demonstrated sustained improvements in weight and waist circumference in the long term.

**Conclusion:**

This study provides evidence for substantial and sustained improvements in self-reported metabolic parameters in people with T2D engaged in a citizen initiative without medical supervision. Initiatives like YLAM offer a promising, accessible and scalable strategy to address the growing burden of lifestyle-related diseases.

**WHAT IS ALREADY KNOWN ON THIS TOPIC**

- Lifestyle interventions can improve and even induce remission in type 2 diabetes (T2D) but often require medical supervision and are difficult to scale up.

**WHAT THIS STUDY ADDS**

- This study demonstrates that a citizen initiative using peer coaching and self-management, without medical supervision, can lead to self-reported improvements in weight, waist circumference, fasting glucose and glycated haemoglobin within 1 year. On average, participants voluntarily reported their measurements every 2 weeks. Almost half of the participants remained engaged for over a year and showed sustained improvements in weight and waist circumference.

**HOW THIS STUDY MIGHT AFFECT RESEARCH, PRACTICE OR POLICY**

- These findings indicate that citizen initiatives such as *Your Lifestyle As Medicine*, which use peer coaching and self-management, offer a promising and novel strategy that can serve as accessible, affordable and scalable additions to professional medical care.

## Introduction

 Type 2 diabetes (T2D) is one of the fastest-growing global health challenges. The International Diabetes Federation estimates that 853 million adults will be living with the disease by 2050, largely due to ageing populations and unhealthy lifestyles.^1^ T2D is frequently accompanied by comorbidities such as cardiovascular diseases, hypertension and obesity, placing an increasing burden on healthcare systems worldwide.^2 3^ Studies have shown that T2D can be prevented through healthy lifestyles.^4^,^6^ Among those already diagnosed, lifestyle modification can even induce remission, which is defined as normalised glucose levels for at least 3 months without glucose-lowering medication.^7^ Although structured interventions such as the DiRECT trial improve metabolic outcomes, they are resource-intensive, reliant on medical supervision and challenging to scale. Moreover, participants frequently relapse when medical supervision ends and unhealthy living environments persist.^8 9^ These limitations highlight the need for sustainable, accessible strategies that support ongoing self-management and long-term behavioural change.

Peer coaching has emerged as a promising strategy to enhance intrinsic motivation and support lifestyle adherence through social connection and shared experience.^10 11^ Several meta-analyses report that peer coaching improves self-management and self-efficacy,^12 13^ which are positively associated with glycaemic control.^14^ However, findings on metabolic parameters, such as glycated haemoglobin (HbA_1_c), body mass index (BMI), low-density lipoprotein cholesterol and systemic blood pressure, remain inconsistent. Most studies evaluated peer coaching within structured, medically supervised settings with limited follow-up.^15^ The long-term engagement and outcomes for people participating in online, community-based diabetes initiatives remain unknown to date.^16^ This reveals a gap in understanding the real-world impact of citizen initiatives operating independently of medical professionals.

This study evaluates the T2D self-management programme of *Your Lifestyle As Medicine* (YLAM), a Dutch citizen initiative promoting lifestyle change through peer coaching and self-management. YLAM was established in 2018 following the initiator’s personal experience of achieving T2D remission through lifestyle change and subsequent termination of glucose-lowering medication. In YLAM’s Facebook communities, people exchange experiential knowledge about lifestyle independent from medical professionals. In addition, educational content is provided through YLAM’s website and webinars with subject-matter experts.^17^ In the Facebook community that specifically targets people with T2D, people reported their self-measured weight, waist circumference, fasting glucose and HbA_1_c on a regular basis. This study evaluates the impact of this self-management approach by analysing these self-reported metabolic parameters and participant engagement through reporting duration and weekly reporting rates. We explored YLAM’s potential as a scalable strategy for sustainable T2D self-management alongside professional medical care.

## Methods

### Study design

The Dutch citizen initiative YLAM has several Facebook communities, including one specifically targeting people with T2D.^18^ Participation and access to all online resources were free of charge. Both the YLAM website and Facebook groups provide information on different strategies to adopt healthier lifestyles. Shared strategies include low-carbohydrate or ketogenic diets, elimination of ultra-processed foods and different types of fasting.^17^ These dietary changes with low carbohydrate load allow participants to reduce or even stop their glucose-lowering medication under the supervision of their medical professionals. In the Facebook communities, members actively support and encourage each other in implementing and maintaining these lifestyle changes. The groups also offer a space for interaction and peer support. Since August 2024, an artificial intelligence chatbot has been available on the website, using existing website content to answer personal questions. YLAM also invited external experts to deliver webinars, providing participants with additional evidence-based information and guidance.

In the YLAM Facebook community specifically for people with T2D, which included approximately 3000 members by the end of the study period in November 2024, a total of 250 people voluntarily reported (weekly) measurements of their weight, waist circumference, fasting glucose and HbA_1_c. These self-reported measurements formed the basis for the analyses presented in this study. This Facebook group was freely accessible to anyone interested in joining, without costs or formal inclusion criteria. The 3000 members include individuals with varying levels of engagement, ranging from one-time visitors and regular followers to actively contributing participants. The 250 data contributors were not selected or recruited by the research team but represent an active subgroup who voluntarily shared their self-measured health data.

Several participants in the initiative act as peer coaches within this specific Facebook group, actively supporting people who reported their measurements by providing feedback on these weekly reports. They also share recipes, lifestyle tips and explain the mechanisms of T2D. Peer coaches were selected by the initiator based on motivation to contribute actively and were granted administrative rights within the Facebook group. Peer coaches received no formal training but relied on their own experiential knowledge. The coaching remained informal and entirely citizen-led. In collaboration with these peer coaches, the initiator of the initiative developed an anonymised database comprising weekly self-reported measurements collected between June 2018 and November 2024. The anonymised dataset included only sex and date of birth and no key or linkage to the original individuals was available.

We used the self-reported data from this database to evaluate metabolic parameters and participant engagement with this self-management approach over time. The cohort has an open, dynamic structure, with continuous enrolment in the cohort until November 2024, when data collection was frozen for the purpose of the present study. As a result, participants contributed their data at different times and for varying durations.

### Study population

The study population consisted of people who reported measurements in the YLAM Facebook community, which is intended for people affected by T2D or interested in its self-management. Participants were included if: (1) they reported at least two measurements of their weight and (2) there was a minimum interval of 3 months between their first and last reported measurements. The initiator’s data were excluded due to their leadership role.

### Data collection

All data were self-measured and reported by participants in the Facebook community for people with T2D. Participants were responsible for conducting their own measurements. Weight was typically recorded using a home scale, and waist circumference was measured using a measuring tape. Measurement instructions were provided and repeated via weekly posts. Fasting glucose values were self-monitored using home glucose meters or continuous glucose monitoring systems (eg, FreeStyle Libre), as is standard practice in diabetes self-management. Alternatively, HbA_1_c values were based on laboratory results obtained through clinical blood tests.

Reported measurements were manually compiled into a database on a weekly basis by the initiator and peer coaches. The dataset was anonymised by replacing participant names with unique codes and was subsequently made available for research purposes to the authors of the present study. Primary outcomes were weight (kg), waist circumference (cm), fasting glucose (mmol/L) and HbA_1_c (mmol/mol). Secondary outcomes were reporting duration (months) and frequency.

Mean relative weight change within the first year was calculated as the percentage difference between the baseline measurement and the final reported weight between months 6 and 12, among participants with data available in that period. Participants’ engagement was evaluated by the duration of reporting and weekly reporting rates per metabolic parameter. The duration of reporting was defined as the period of time between the first and the last reported measurement. Weekly reporting rates reflected the frequency of weekly reported measurements relative to the total number of weeks during a participant’s reporting period.

### Data analysis

Longitudinal changes in these metabolic parameters were analysed using linear mixed models with random intercepts to account for intraindividual correlation due to repeated measurements. Time since the first reported measurement was categorised into predefined intervals: baseline (defined as the first measurement and the subsequent 2 weeks), 2 weeks to 3 months, 3–6 months, 6–12 months, 1–2 years, 2–3 years and more than 3 years. These intervals were included as fixed effects to assess changes in weight, waist circumference, fasting glucose and HbA_1_c over time. Models were adjusted for age, reporting duration and baseline values. Analyses were stratified by sex and conducted in R software (V.4.4.1; R Core Team, Vienna, Austria, 2025) using the *lmerTest* package (V.3.1.3).

### Ethical considerations

All data used in this study were anonymised by the initiator and several peer coaches prior to analysis. Participation in YLAM is entirely voluntary. On joining the Facebook community, participants were presented with a clear statement informing them that their self-reported data could be used for research purposes and were asked to provide active confirmation of their consent.

## Results

### Study population

Of the 250 participants, 18 were excluded for reporting only one measurement (n=12), reporting for a period of less than 3 months (n=5) or being the initiator (n=1). The 232 included participants provided 31 621 data points for analyses, including 10 965 weight measurements, 10 256 waist circumference measurements, 9636 fasting glucose measurements and 764 HbA_1_c measurements. All 232 participants reported their weight, and their first reported measurement defined the start of their participation. Waist circumference was reported by 230 participants, of whom 203 provided a measurement within the baseline period. Fasting glucose measurements were reported by 206 participants, with 170 reporting within baseline. Lastly, HbA_1_c measurements were reported by 166 participants, of whom 104 reported within the baseline period. The study population was predominantly women (68%). At baseline, mean age was 56.8 (*SD* 9.4) years, and mean BMI was 31.2 (SD 5.7) kg/m^2^ (table 1).

**Table 1:** Participants’ characteristics at baseline

	Total (n=232)	Women (n=158)	Men (n=74)
Age at baseline (years)	56.8 (9.4)	56.3 (9.2)	57.8 (9.9)
BMI at baseline (kg/m^2^)	31.2 (5.7)	31.5 (5.6)	30.7 (6.0)
Duration of reporting (months)	11.2 (4.6–26.5)	11.2 (4.6–26.8)	8.3 (4.6–22.7)
Weight at baseline (kg)	92.7 (19.3)	88.6 (16.3)	101.3 (22.3)
Waist circumference at baseline (cm)*	107.7 (13.8)	106.8 (13.9)	109.8 (13.7)
Fasting glucose at baseline (mmol/L)†	8.5 (3.0)	8.5 (3.1)	8.5 (2.8)
HbA_1_c at baseline (mmol/mol)‡	60.9 (16.9)	61.4 (17.3)	59.7 (15.9)

Data are presented as mean (SD) or median (IQR), depending on distribution.

* Data available from a subset of 203 participants (141 women, 62 men).

† Data available from a subset of 170 participants (119 women, 51 men).

‡ Data available from a subset of 104 participants (75 women, 29 men).

BMI, body mass index; HbA_1_c, glycated haemoglobin; n, number of participants.

### Participants’ engagement

The overall median duration of reporting was 11.2 months (IQR 4.6–26.5), with women reporting for 11.2 months (IQR 4.6–26.8) and men for 8.3 months (IQR 4.6–22.7). Of all 232 participants, 103 (44%) remained engaged for longer than a year. In the last 3 months of the study period (between 28 August and 28 November 2024), 46 participants (20%) had reported at least one measurement to the online community. Of all participants, the weekly reporting rate was on average 59% for weight, 55% for waist circumference and 52% for fasting glucose. HbA_1_c had a 4% weekly and 49% 12-weekly reporting rate, which aligns with its standard measurement interval of approximately 3 months.

### Assessment of changes in metabolic parameters

**Figure 1:**
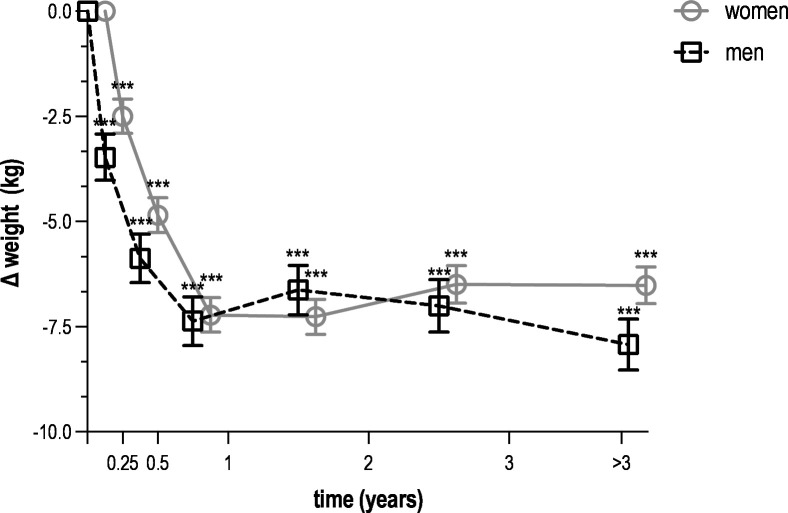
Mean change in weight over time among *Your Lifestyle As Medicine* participants, stratified by sex (women in grey, men in black). Values are presented as means with 95% CIs (error bars) for each time interval. Data include all 232 participants who reported at least two weight measurements over a period of 3 months or more. The number of participants varied across time intervals due to rolling enrolment and dropout in this dynamic cohort. Time intervals were defined as follows: baseline (<2 weeks, n=232), 2 weeks to 3 months (n=204), 3–6 months (n=178), 6–12 months (n=141), 1–2 years (n=97), 2–3 years (n=52) and more than 3 years (n=42). Statistical significance is indicated by asterisks (***p<0.001) for within-sex comparisons with baseline values.

**Figure 2:**
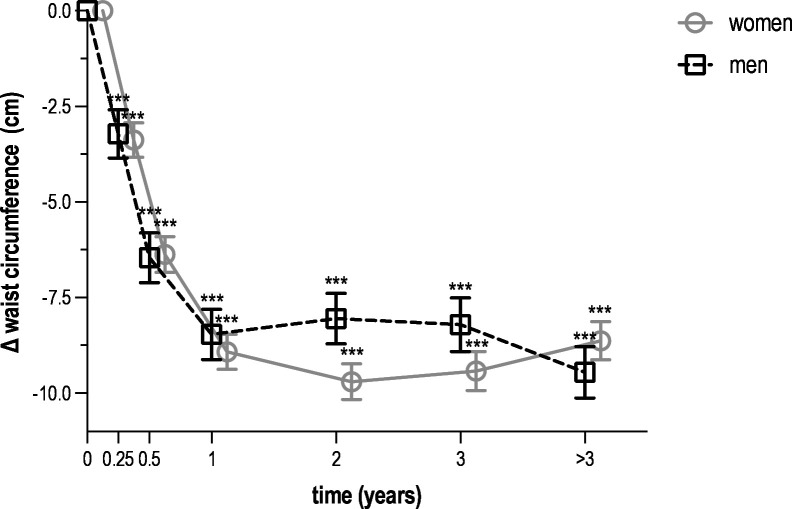
Mean change in waist circumference over time among *Your Lifestyle As Medicine* participants, stratified by sex (women in grey, men in black). Values are presented as means with 95% CIs (error bars) for each time interval. Data include all 230 participants who reported at least two measurements over a period of 3 months or more. The number of participants varied across time intervals due to rolling enrolment and dropout in this dynamic cohort. Time intervals were defined as follows: baseline (<2 weeks, n=203), 2 weeks to 3 months (n=202), 3–6 months (n=174), 6–12 months (n=140), 1–2 years (n=96), 2–3 years (n=50) and more than 3 years (n=40). Statistical significance is indicated by asterisks (***p<0.001) for within-sex comparisons with baseline values.

**Figure 3:**
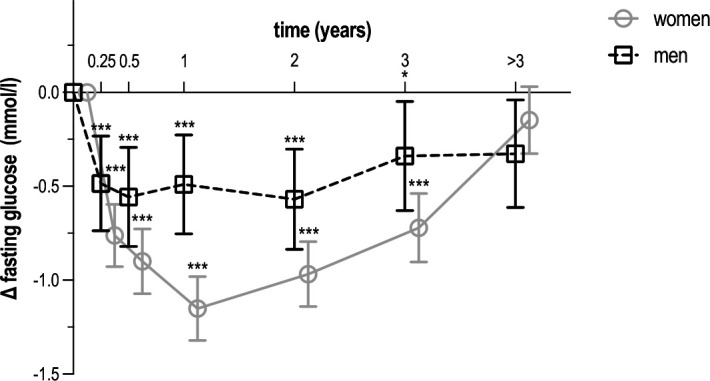
Mean change in fasting glucose over time among *Your Lifestyle As Medicine* participants, stratified by sex (women in grey, men in black). Values are presented as means with 95% CIs (error bars) for each time interval. Data include all 206 participants who reported at least two measurements over a period of 3 months or more. The number of participants varied across time intervals due to rolling enrolment and dropout in this dynamic cohort. Time intervals were defined as follows: baseline (<2 weeks, n=170), 2 weeks to 3 months (n=174), 3–6 months (n=154), 6–12 months (n=127), 1–2 years (n=91), 2–3 years (n=49) and more than 3 years (n=42). Statistical significance is indicated by asterisks (*p<0.05; ***p<0.001) for within-sex comparisons with baseline values.

**Figure 4:**
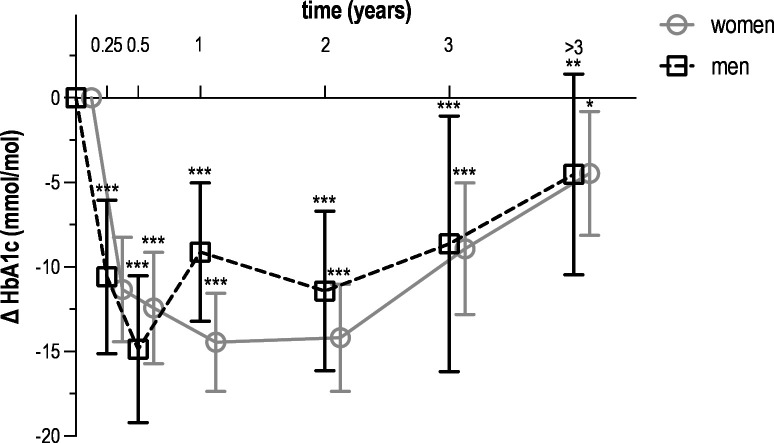
Mean change in HbA_1_c over time among *Your Lifestyle As Medicine* participants, stratified by sex (women in grey, men in black). Values are presented as means with 95% CIs (error bars) for each time interval. Data include all 166 participants who reported at least two measurements over a period of 3 months or more. The number of participants varied across time intervals due to rolling enrolment and dropout in this dynamic cohort. Time intervals were defined as follows: baseline (<2 weeks, n=104), 2 weeks to 3 months (n=93), 3–6 months (n=86), 6–12 months (n=96), 1–2 years (n=70), 2–3 years (n=44) and more than 3 years (n=31). Statistical significance is indicated by asterisks (*p<0.05; **p<0.01; ***p<0.001) for within-sex comparisons with baseline values. HbA_1_c, glycated haemoglobin.

All metabolic parameters improved during the first year. More specifically, weight decreased by 7.2 kg in women (95% CI –7.6 to –6.8) and by 7.4 kg in men (95% CI –8.0 to –6.8) (figure 1). Among participants with available weight measurements at both baseline and 6–12 months, this corresponded to a mean relative weight loss of 9.0% (SD 7.7) in women and 8.6% (SD 6.5) in men. Waist circumference decreased by 8.9 cm in women (95% CI –9.4 to –8.5) and by 8.5 cm in men (95% CI –9.1 to –7.8) (figure 2). Fasting glucose decreased by 1.15 mmol/L in women (95% CI –1.32 to –0.98) and by 0.49 mmol/L in men (95% CI –0.75 to –0.23) (figure 3). HbA_1_c decreased by 14.5 mmol/mol in women (95% CI –17.4 to –11.6) and by 9.1 mmol/mol in men (95% CI –13.2 to –5.0) (figure 4). Among long-term reporters, decreases in self-reported metabolic parameters were sustained relative to baseline values. Among the 42 participants who reported weight measurements more than 3 years after baseline, mean weight was 6.5 kg lower in women (95% CI –7.0 to –6.1) and 7.9 kg lower in men (95% CI –8.5 to –7.3). Among the 40 participants who reported waist circumference measurements more than 3 years after baseline, mean waist circumference had decreased by 8.6 cm in women (95% CI –9.1 to –8.1) and by 9.5 cm in men (95% CI –10.1 to –8.8). Among the 42 participants who reported fasting glucose measurements more than 3 years after baseline, mean levels had decreased by 0.15 mmol/L in women (95% CI –0.33 to 0.03) and by 0.33 mmol/L in men (95% CI –0.61 to –0.04). Among the 31 participants who reported HbA_1_c measurements more than 3 years after baseline, mean HbA_1_c levels had decreased by 4.5 mmol/mol in women (95% CI –8.1 to –0.8) and men (95% CI –10.5 to 1.4). Online supplemental Tables S1a–S4b provide numerical values for figures1^4^.

## Discussion

This study demonstrated that a citizen initiative using peer coaching and self-management without medical supervision can support people with T2D in achieving and maintaining clinically relevant improvements in metabolic health. Participants in the self-management approach of YLAM experienced substantial and sustained reductions in weight, waist circumference, fasting glucose and HbA_1_c. On average, the self-reported improvements were predominantly observed in the first year. These improvements were sustained, although a gradual increase in fasting glucose and HbA_1_c was observed, particularly in women. Notably, nearly half of all participants remained engaged for over a year, with sustained improvements in weight and waist circumference over time.

### Interpretation in context

These findings align with prior research demonstrating the potential of lifestyle interventions to improve metabolic outcomes^19^,^21^ and induce T2D remission.^6 20^ Peer coaching has emerged as a promising strategy to support these changes, improving self-efficacy and self-management skills through shared lived experience.^12 13^ However, these often involved intensive professional supervision within clinical frameworks and short follow-up periods.^6 8 12 13 16^ This limits insight into the functioning of peer support in autonomous, community-based settings over extended timeframes without the involvement of healthcare professionals, such as the online YLAM initiative.

Online communities have been recognised as valuable platforms for diabetes self-management, as they offer continuous access to (peer) support.^16^ However, most studies in this area report limited sample sizes, limited follow-up or focus on self-management rather than metabolic outcomes.^22^,^24^ Our study adds to these findings by demonstrating favourable metabolic trends in a real-world online cohort and by providing data not commonly collected on long-term engagement in such settings. To our knowledge, no similarly designed citizen initiative without the involvement of medical supervision has reported such substantial reductions in weight and waist circumference. These findings suggest that initiatives using peer coaching and self-management in an online community may effectively complement conventional approaches to diabetes care.

Insights from the initiator and peer coaches may help explain long-term participant engagement. According to the initiator and peer coaches, a key factor appeared to be the use of experiential knowledge, which enabled peer coaches to act as relatable role models. Participants may feel more motivated and understood when supported by others who have faced similar challenges. Such support may help build trust, enhance social support and foster a sense of community ownership, as also described by Verma *et al*.^13^ While these impressions remain speculative in the context of this study, they highlight the potential added value of peer-led initiatives and underscore the importance of further research into their mechanisms of action.

Social media platforms like Facebook have increasingly been used to support self-management in people with chronic conditions such as T2D. Previous studies have shown that Facebook-based communities can foster information exchange, emotional support and a sense of belonging.^25 26^ Interactions in such groups often centre around shared lived experiences, and peers may contribute to positive social influence and encourage health-promoting behaviours. Our findings add to this literature by showing that a large, unsupervised Facebook community, maintained by peer volunteers, can facilitate long-term engagement and measurable improvements in self-reported metabolic outcomes. Although this study did not explicitly evaluate the digital platform as an intervention, the success of the YLAM community supports the potential of social media to enable citizen initiatives for promoting health. This aligns with earlier work demonstrating that Facebook can be an effective platform for delivering health interventions and increasing engagement and self-efficacy.^27^ In this light, citizen initiatives such as YLAM can also be seen as a form of citizen science, where public engagement supports data sharing, peer learning and health knowledge creation in real-world contexts.

Sex-specific differences were also observed, particularly in glycaemic outcomes. While overall patterns in weight and waist circumference were largely similar between women and men, some sex differences emerged in glycaemic outcomes. Women showed greater reductions in fasting glucose, with more subtle differences in HbA_1_c. These findings should be interpreted with caution, as glucose regulation is strongly influenced by medication use. However, information on medication was not available in this study. Additionally, women experienced a slightly larger relative weight loss within the first year (8.1% vs 7.3%), which may have indirectly contributed to their fasting glucose improvements. Lastly, a previous study suggested that women may be more engaged in self-monitoring and health-promoting behaviours, which could partially explain their higher participation rates and potentially stronger early metabolic responses.^28^

### Strengths and limitations

This study offers valuable insights into how citizen initiatives may function as additions to professional medical care. Its strengths include a real-world dataset of more than 30 000 self-reported metabolic measurements from over 200 people, capturing engagement over multiple years. By evaluating this citizen initiative, which had not primarily been set up for research purposes, the study reflects on real-life participants’ engagement, adding a unique perspective to the field of diabetes care.

Nonetheless, several limitations must be acknowledged. First, most data were self-measured, and all data were self-reported, introducing two sources of potential bias. Participants measured their weight, waist circumference and fasting glucose, using home devices. While these measurements are in line with common practices in diabetes self-management, they may still introduce measurement error due to inconsistent techniques or device inaccuracies. For HbA_1_c, participants reported values obtained from clinical blood tests performed by their treating physicians. As data were self-reported and unverifiable by the research team, we cannot exclude the possibility that some participants relied on alternative sources or reported inaccurately. Together, the self-measurement and self-reporting processes may have affected data accuracy. Also, the research team had no role in data collection, and no access to key clinical variables, such as medication use, comorbidities or T2D diagnosis confirmations. Consequently, it remains unclear to what extent changes in medication, such as insulin or glucose-lowering medication, may have contributed to the self-reported changes in metabolic parameters. Additionally, motivations for joining and remaining engaged in the community remain unknown, since no data on psychosocial characteristics were collected.

Due to the open and dynamic nature of the study, participant dropout was difficult to interpret. Some people may have stopped reporting after achieving their health goals, while others may have disengaged due to a lack of progress or declining motivation. Only 17 participants dropped out within 3 months, most of whom reported only one measurement. However, the reasons for disengagement remain unknown, and no qualitative or follow-up data were available to clarify this. In this context, selection and reporting bias are important limitations. Since data sharing was voluntary and non-anonymous within the Facebook community, participants with less favourable outcomes may have been less likely to continue reporting or may have under-reported their results. As a result, the observed improvements may reflect a particularly motivated and successful subset of the community.

Despite these uncertainties regarding disengagement, nearly half (44%) of all participants remained engaged for more than a year. Because the study employed a dynamic cohort with rolling enrolment, reporting duration was likely underestimated, as some participants were probably still active beyond the data freeze in November 2024. This sustained level of voluntary participation is particularly notable given that previous peer support studies often report follow-up periods of only several weeks to 6 months.^16^ These findings suggest that public social platforms may offer a feasible environment for long-term self-monitoring and engagement, although selective dropout remains a concern.

Finally, due to the observational nature of this study, it lacks a control group, which limits the ability to infer causality. Participants were self-selected, digitally literate and likely highly motivated to improve their health. Also, participants not only engaged in regular self-monitoring but chose to share their data openly with the community, which may exclude those who prefer to track privately. This limits the generalisability of the findings to broader T2D populations, especially those with less digital literacy or motivation. Still, this study indicates that there is a group of people with T2D for whom citizen initiatives offer substantial improvements in metabolic parameters. Another consideration is that the study sample was disproportionately composed of women (158 women vs 74 men). The smaller number of men contributed to greater variability and wider confidence intervals. We performed stratified analyses to mitigate the impact of sex imbalance on the generalisability of our findings to the broader T2D population, given the previously reported sex-specific differences in health behaviour and pathophysiology.^28^,^30^

Taken together, these limitations underscore the need to interpret the findings cautiously. Given the uncontrolled, observational design, no conclusions reflecting causality can be made about the impact of the citizen initiative on the observed improvements. Rather than demonstrating efficacy, this study offers real-world insights into what occurred among a motivated group of individuals with T2D who voluntarily engaged in peer-led self-management.

### Risks

While peer coaching offers clear benefits, it also poses potential risks. Peer coaches are not professionals, and their advice may not always align with clinical guidelines. In particular, adjusting diet or activity levels without concurrent medication review can lead to adverse effects, such as hypoglycaemia.^31 32^ Because the research team had no access to clinical records or medication data, and no clinical monitoring was in place, we were unable to assess whether any participants experienced harm as a result of lifestyle changes. If citizen initiatives like YLAM are to be more broadly adopted or integrated into care systems, safeguards should be considered, such as clearer referral pathways or partnerships with healthcare providers. When appropriately aligned, experiential knowledge of peers and professional expertise could complement one another to enhance safety and effectiveness.

### Future research and implications

Future research should focus on who is willing to participate, why people stay engaged and what barriers keep others from participating. This will be critical for broadening accessibility and reach. Despite its methodological limitations, this study underscores the importance of exploring sustainable, citizen-driven strategies to manage lifestyle-related diseases. Citizen initiatives such as YLAM may represent an accessible, affordable and scalable addition to professional medical care. These initiatives could particularly be helpful to alleviate pressure in resource-limited settings by promoting self-management. Policymakers and healthcare providers should consider supporting or collaborating with such health initiatives.

In conclusion, citizen initiatives using peer coaching and self-management could support people with T2D in achieving and maintaining substantial, clinically relevant improvements in metabolic health.

## Supplementary material

10.1136/bmjnph-2025-001362 online supplemental table 1

## Data Availability

All data relevant to the study are included in the article or uploaded as supplementary information.
