# Supplementary material for: Your Lifestyle As Medicine: the impact of a citizen initiative for people with type 2 diabetes using peer coaching and self-management
Source: BMJ Nutr Prev Health. 2025 Nov 5;8(2):e001362. doi: 10.1136/bmjnph-2025-001362 (PMC12772579; doi:10.1136/bmjnph-2025-001362)
Supplement: online supplemental table 1 [file bmjnph-8-2-s001.pdf]

Supplementary Table S1a. Mean change in weight over time in women (*n* = 158)

	n	**Δ mean (kg)**	**95% CI**	***p*-value**
Baseline (<2 weeks)	158	0	-	*Ref*
2 weeks to 3 months	136	–2.5	–2.9 to –2.1	<0.001
3 to 6 months	121	–4.8	–5.3 to –4.4	<0.001
6 to 12 months	100	–7.2	–7.6 to –6.8	<0.001
1 to 2 years	70	–7.3	–7.7 to –6.9	<0.001
2 to 3 years	38	–6.5	–6.9 to –6.1	<0.001
>3 years	30	–6.5	–7.0 to –6.1	<0.001

Supplementary Table S1b. Mean change in weight over time in men (*n* = 74)

	n	**Δ mean (kg)**	**95% CI**	***p*-value**
Baseline (<2 weeks)	74	0	-	*Ref*
2 weeks to 3 months	68	–3.5	–4.0 to –2.9	<0.001
3 to 6 months	57	–5.9	–6.5 to –5.3	<0.001
6 to 12 months	41	–7.4	–8.0 to –6.8	<0.001
1 to 2 years	27	–6.6	–7.2 to –6.0	<0.001
2 to 3 years	14	–7.0	–7.6 to –6.4	<0.001
>3 years	12	–7.9	–8.5 to –7.3	<0.001

Supplementary Table S2a. Mean change in waist circumference over time in women (n = 156)

	n	**Δ mean (cm)**	**95% CI**	***p*-value**
Baseline (<2 weeks)	141	0	-	*Ref*
2 weeks to 3 months	135	–3.4	–3.8 to –2.9	<0.001
3 to 6 months	117	–6.4	–6.8 to –5.9	<0.001
6 to 12 months	99	–8.9	–9.4 to –8.5	<0.001
1 to 2 years	70	–9.7	–10.1 to –9.2	<0.001
2 to 3 years	37	–9.4	–9.9 to –8.9	<0.001
>3 years	28	–8.6	–9.1 to –8.1	<0.001

Supplementary Table S2b. Mean change in waist circumference over time in men (n = 74)

	n	**Δ mean (cm)**	**95% CI**	***p*-value**
Baseline (<2 weeks)	62	0	-	*Ref*
2 weeks to 3 months	67	–3.2	–3.9 to –2.6	<0.001
3 to 6 months	57	–6.5	–7.1 to –5.8	<0.001
6 to 12 months	41	–8.5	–9.1 to –7.8	<0.001
1 to 2 years	26	–8.0	–8.7 to –7.4	<0.001
2 to 3 years	13	–8.2	–8.9 to –7.5	<0.001
>3 years	12	–9.5	–10.1 to –8.8	<0.001

Supplementary Table S3a. Mean change in fasting glucose over time in women (*n* = 138)

	n	**Δ mean (mmol/L)**	**95% CI**	***p*-value**
Baseline (<2 weeks)	119	0	-	*Ref*
2 weeks to 3 months	115	–0.76	–0.93 to –0.60	<0.001
3 to 6 months	103	–0.90	–1.07 to –0.73	<0.001
6 to 12 months	89	–1.15	–1.32 to –0.98	<0.001
1 to 2 years	65	–0.97	–1.14 to –0.80	<0.001
2 to 3 years	36	–0.72	–0.90 to –0.54	<0.001
>3 years	30	–0.15	–0.33 to 0.03	0.104

Supplementary Table S3b. Mean change in fasting glucose over time in men (*n* = 68)

	n	**Δ mean (mmol/L)**	**95% CI**	***p*-value**
Baseline (<2 weeks)	51	0	-	*Ref*
2 weeks to 3 months	59	–0.49	–0.74 to –0.23	<0.001
3 to 6 months	51	–0.56	–0.82 to –0.29	<0.001
6 to 12 months	38	–0.49	–0.75 to –0.23	<0.001
1 to 2 years	26	–0.57	–0.84 to –0.30	<0.001
2 to 3 years	13	–0.34	–0.63 to –0.05	0.020
>3 years	12	–0.33	–0.61 to –0.04	0.822

Supplementary Table S4a. Mean change in HbA_1c over time in women (*n* = 115)

	n	**Δ mean (mmol/mol)**	**95% CI**	***p*-value**
Baseline (<2 weeks)	75	0	-	*Ref*
2 weeks to 3 months	62	–11.3	–14.4 to –8.3	<0.001
3 to 6 months	60	–12.4	–15.7 to –9.1	<0.001
6 to 12 months	70	–14.5	–17.4 to –11.6	<0.001
1 to 2 years	53	–14.2	–17.4 to –11.0	<0.001
2 to 3 years	27	–8.9	–12.8 to –5.0	<0.001
>3 years	22	–4.5	–8.1 to –0.8	0.012

Supplementary Table S4b. Mean change in HbA_1c over time in men (*n* = 51)

	n	**Δ mean (mmol/mol)**	**95% CI**	***p*-value**
Baseline (<2 weeks)	29	0	-	*Ref*
2 weeks to 3 months	31	–10.6	–15.1 to –6.1	<0.001
3 to 6 months	26	–14.9	–19.2 to –10.5	<0.001
6 to 12 months	26	–9.1	–13.2 to –5.0	<0.001
1 to 2 years	17	–11.4	–16.1 to –6.7	<0.001
2 to 3 years	8	–8.6	–16.2 to –1.1	<0.001
>3 years	9	–4.5	–10.5 to 1.4	0.008

Notes: Values represent mean changes from baseline for each time interval, with 95% confidence intervals (95%CI). Baseline is defined as the first reported measurement and the subsequent two weeks. The *p*-values reflect within-group comparisons with baseline, based on linear mixed-model analyses adjusted for age, reporting duration, and baseline value of the respective metabolic parameter. Sample sizes (*n*) per time interval may vary due to rolling enrolment and dropout in this dynamic cohort. This note applies to Supplementary Tables S1a–S4b.
